# Optimizing Resource Allocation in a Cowpea (*Vigna unguiculata* L. Walp.) Landrace Through Whole-Plant Field Phenotyping and Non-stop Selection to Sustain Increased Genetic Gain Across a Decade

**DOI:** 10.3389/fpls.2019.00949

**Published:** 2019-08-07

**Authors:** Michalis Omirou, Ioannis M. Ioannides, Dionysia A. Fasoula

**Affiliations:** ^1^Department of Agrobiotechnology, Agricultural Research Institute, Nicosia, Cyprus; ^2^Department of Plant Breeding, Agricultural Research Institute, Nicosia, Cyprus

**Keywords:** prognostic breeding, Honeycomb selection designs, phenotyping equations, interplant competition, G×E interaction, climate change, spatial heterogeneity, root phenotyping

## Abstract

Cowpea is a warm-season legume, often characterized as an orphan or underutilized crop, with great future potential, particularly under the global change. A traditional cowpea landrace in Cyprus is highly valued for fresh pod consumption in the local cuisine. In order to improve the yield potential of the landrace, the long-term response to direct selection for fresh pod yield and the associated changes in fodder and root biomass were investigated in a variety of fertility regimes under real field conditions. The non-stop selection process employed comprehensive pod, fodder, and root phenotyping at the level of the individual plant and resulted in the creation of a range of highly improved sibling lines with differential adaptation to micro-environments and with an improved ratio of pod to shoot and root biomass. The average rate of increase per year for fresh pod yield is at the level of 180 g per plant despite the relatively narrow genetic base of a single landrace and it is seemingly inexhaustible testifying to the great plasticity of the cowpea genome and the potential of the methodology to capture it. The corresponding high genetic gain was also confirmed under dense stands where the difference in pod yield between the best selection and the control amounted to 31.37%. Thus, the new focus apart from the simple variety maintenance should also include the continuous improvement and exploitation of micro-adaptation processes specific for individual fields that allow quick responses to environmental and climatic changes. This work presents also a novel approach to the multiple challenges encountered in root phenotyping and a method to meaningfully associate it with whole-plant performance in field conditions.

## Introduction

Cowpea (*Vigna unguiculata* L. Walp.) is a warm-season legume, traditionally very important as a food staple and source of fodder for the African continent, India, the Americas, and other semi-arid regions (Timko and Singh, 2008), increasingly gaining importance in other parts of the world, including Europe. As cowpea is well adapted to drought-prone areas and has higher heat tolerance compared to other species (Lucas et al., 2013; Spriggs et al., 2018), its cultivation boundaries are expected to be shifted away from the tropics and sub-tropical areas in response to climate change (Draghici et al., 2016) and its status as a valuable crop is being elevated.

This trend is further significant for Europe where a shortage in crop protein sources has recently been addressed (Schreuder and Visser, 2014) and the significance of legumes is persistently being highlighted in recent years (Foyer et al., 2016). As a legume, cowpea provides an inexpensive source of protein, possessing a tremendous potential to contribute to the alleviation of protein deficiency. Its nutritious grains contain about 21–31% protein and 64% carbohydrate (Bressani, 1985; Timko and Singh, 2008; Gerrano et al., 2019), while it has been reported that cowpea fodder could contain up to 18.6 g protein per 100 g dry weight (Duke, 1990). In addition, cowpea contributes to the sustainability of cropping systems and improvement of soil fertility in marginal lands by providing ground cover and plant residues, nitrogen fixation, and weed suppression (Inaizumi et al., 1999). Cowpea is highly self-pollinated with experimental work suggesting that the pollination process in cultivated cowpeas is complete before the flower opens, while the occasional cross-pollination has been estimated to <1 or up to 2% (OECD, 2016).

**FIGURE 1:**
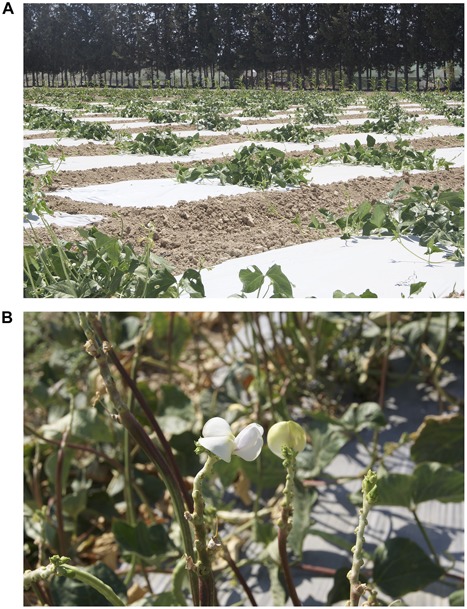
Selection field of the local cowpea landrace “Argaka.” It is arranged as the Honeycomb selection design D19 for evaluation of 19 different entries at ultra-wide distances that exclude any interplant interference with the equal sharing of resources and permit the unhindered expression of the phenotype **(A)**. Cowpea plants of the “Argaka” landrace in bloom after several consecutive harvests of fresh pods. The stem projections below the flowers indicate the attachment points of already harvested pods **(B)**.

The geographic location of Cyprus in the southeastern Mediterranean area provides a rather ideal environment for the cultivation of cowpea. Cowpea landraces occupy an important place in local cuisine, consumed both as immature fresh pods and as dry beans, whereas traditional animal farmers appreciate their fodder, usually what remains after the pod or grain consumption by humans. Cyprus landraces exhibit a highly prostrate habit with trailing plants possessing a large above ground biomass (Figure 1A) and are distinguished in two main types by their capacity to produce fresh pods in multiple (5–7), sequential harvests during spring and early summer (Figure 1B) or by their capacity to produce dry beans once or twice in early fall. Due to the plant morphology, the harvest of fresh or dry pods is performed manually.

One of the most widely cultivated landraces in Cyprus is broadly called “Argaka” after a village bearing the same name in the west of the country and it is used for fresh pod production. In an effort to investigate reported productivity differences among various farmers, we undertook a long-term study of this landrace spanning the period 2009–2017.

It has been discovered (Fasoula, 1990) that when varieties are propagated under the dense stands of commercial production regimes in order to produce seed for the next sowing season, the negative correlation between yielding and competitive ability, measured for the first time, favors plants that possess lower yielding, but higher competitive ability. Those plants are propagated preferentially at the expense of higher yielding plants with lower competitive ability. As this common propagation practice under dense stands and visual selection is repeated over successive years, it gradually leads to the tangible degeneration of the variety and to the subsequent increase in non-uniformity within (Fasoula, 2011; Fasoula and Omirou, 2018b). The notion of the negative correlation between yielding and competitive ability has been further corroborated in cowpea (Kumar and Mishra, 2000).

As an antidote to the above, the concept of non-stop selection (Fasoula and Fasoula, 2000; Fasoula, 2012) has been proposed and successfully implemented in additional crops for both yield and quality traits (Fasoula and Boerma, 2005). The concept entails the year-to-year propagation of plants destined to produce seeds for the next sowing season under conditions that exclude any interplant interference (Fasoula and Fasoula, 1997), i.e., ultra-wide plant-to-plant distances and thorough eradication of weeds, as well as under conditions that effectively account for the masking effects of field heterogeneity in the selection process through the use of the Honeycomb selection designs (Fasoulas and Fasoula, 1995). No visual selection occurs at any stage.

When the practice of non-stop selection is combined with the principles of the prognostic breeding paradigm (Fasoula, 2013) the results are quite profound (Greveniotis and Fasoula, 2016). In prognostic breeding, the entries after the first cycle of selection consist of sibling families or lines, representing a group of plants having the same mother. This permits the simultaneous evaluation of individual plants on the basis of the two components of the crop yield potential, i.e., the plant yield potential and the stability index (SI), and greatly increases the efficiency of selection, since plant yield and stability are assessed concurrently in the same generation through each plant’s siblings, rather than through progeny testing in successive generations and years.

The aim of this study has been to investigate the long-term results of the application of non-stop selection and the prognostic breeding paradigm to the productivity and plant morphology of the local cowpea landrace “Argaka” exploring the limits of genetic progress that can be achieved within the relatively narrow genetic base of a single landrace of a highly selfing species and the possibility to continuously isolate superior genotypes with improved adaptation targeted at each specific micro-environment representing farmers’ fields.

## Materials and Methods

### Site Characteristics and Selection Principles Outline

The study was initiated in May 2009 with seeds from the local cowpea landrace “Argaka” derived from farmers of the west Cyprus village bearing the same name. The landrace is characterized by its prostrate habit and the capacity to flower in multiple flushes producing fresh pods in five to seven sequential harvests performed by hand during a period of about 3 months in late spring and summer (Figure 1B). All trials (2009–2017) were conducted at the Zygi Experimental Station (34°44′30″N 33°19′42.5″E) of the Agricultural Research Institute. The field soil consisted of 22% sand, 36% silt, and 42% clay with pH (1:2.5 soil:water slurry) value of 8.2. The total organic C (Walkley–Black method) and N (Kjeldahl method) was 1.62 and 0.092%, respectively. During all years, plants were transplanted to the field at the two-leaf stage. The practice of transplantation is commonly used by local farmers and was therefore adopted for all trials. Each plant was individually irrigated through drip irrigation according to standard practices, while plant nutrients were applied by fertigation on a weekly basis depending on the irrigation needs and the growth stage of the plants using 19-19-19 as fertilizer except during 2009, where only basal fertilization was applied using 20-20-20 as fertilizer. Pest control was achieved following standard farming practices in the area and according to pest populations and disease incidences.

The arrangement of individual plants in the field (Figure 1A) followed one of the multiple Honeycomb selection designs (Fasoulas and Fasoula, 1995), as described under each year. During the first 3 years (2009–2011) the distance between the individual plants was 1 m within row and 0.9 m between rows to exclude any interplant interference. However, during the subsequent years and because of the trailing habit of the landrace, the distance between individual plants was increased to 1.8 m between rows and 2 m on the row. This distance facilitated pod harvest and was also most appropriate to perform individual root biomass measurements after Year 4. Each trial included a design code assigned to the control, i.e., bulked remnant seed from the original farmer source, except for years 2011 and 2017, when the control included progenies of a single control plant of the previous year and not the remnant bulk. The design code numbers assigned to sibling families during each year are independent of those used in other years, i.e., the line with design code 1 during 2010 is not the same with lines assigned the code 1 in subsequent years.

Selection of superior plants was based on the values of the plant phenotyping or plant prognostic equation, pPE, of fresh pod weight:

(1) $$\text{pPE}={\left(x/\bar{x_{r}}\right)}^{2}.{\left(\bar{x}/s\right)}^{2}$$

**FIGURE 2:**
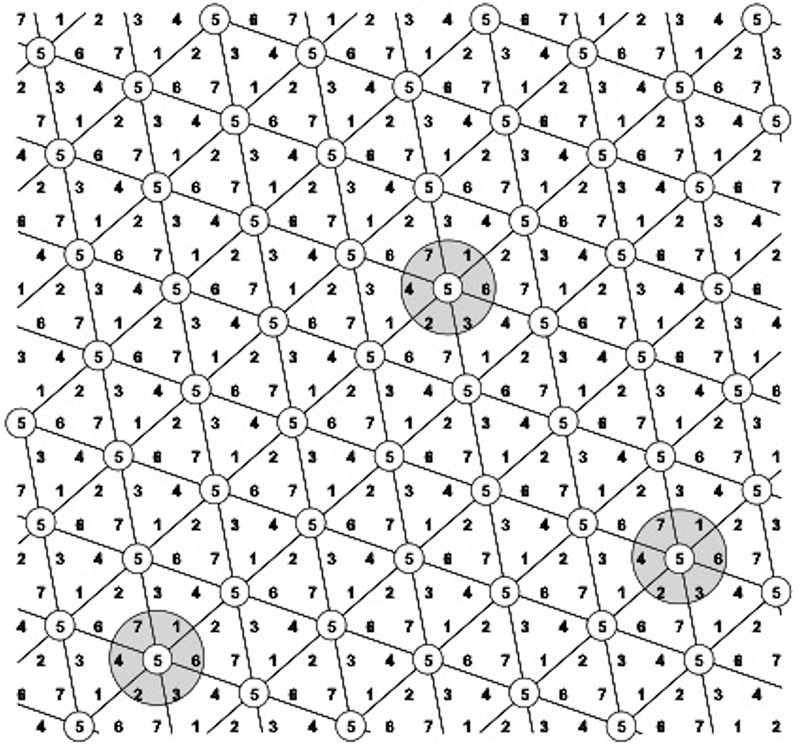
The D7 Honeycomb selection design for the evaluation of seven different entries with design codes 1–7. Every plant belongs to the center of a complete and moving replicate, exemplified by the gray circles (moving complete blocks – MCB) across all field gradients. Plants with the same code numbers belong to the same entry and form a symmetrical and moving complete triangular grid across the field.

**FIGURE 3:**
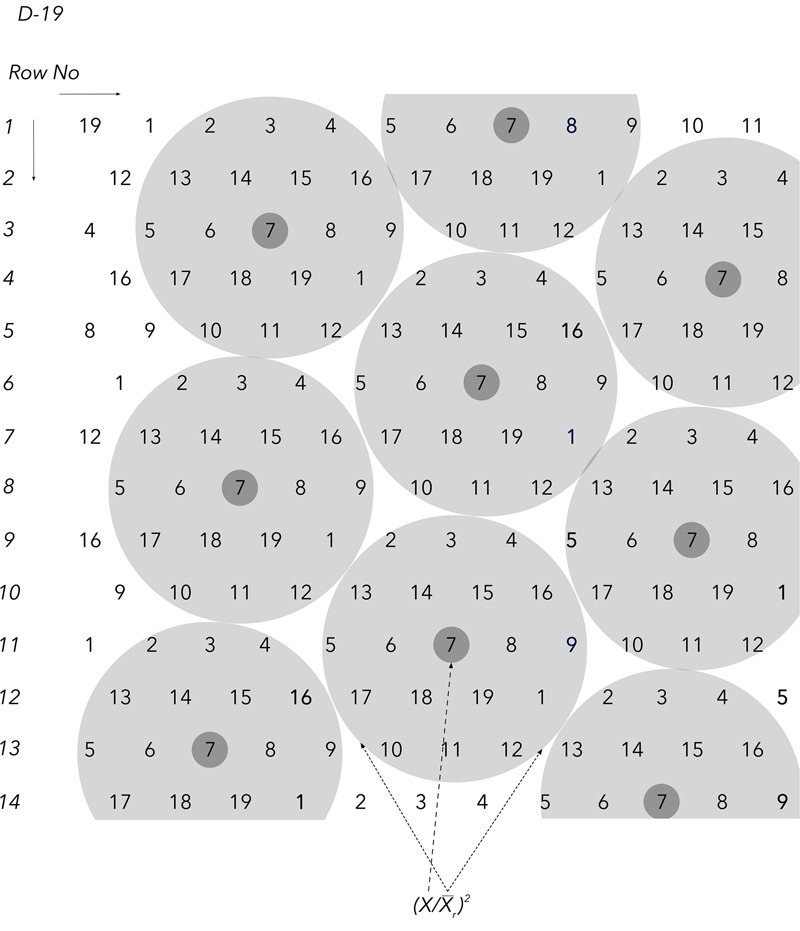
The D19 Honeycomb selection design for the evaluation of 19 different entries with design codes 1–19. The derivation of the first component of the plant phenotyping equation and the principle of the moving complete block (complete and moving replicates) are exemplified with entry code 8 in the center. Notably, the unique equation value that characterizes each individual plant in all Honeycomb designs encompasses the stability of yield across environments through the stability index (SI).

**FIGURE 4:**
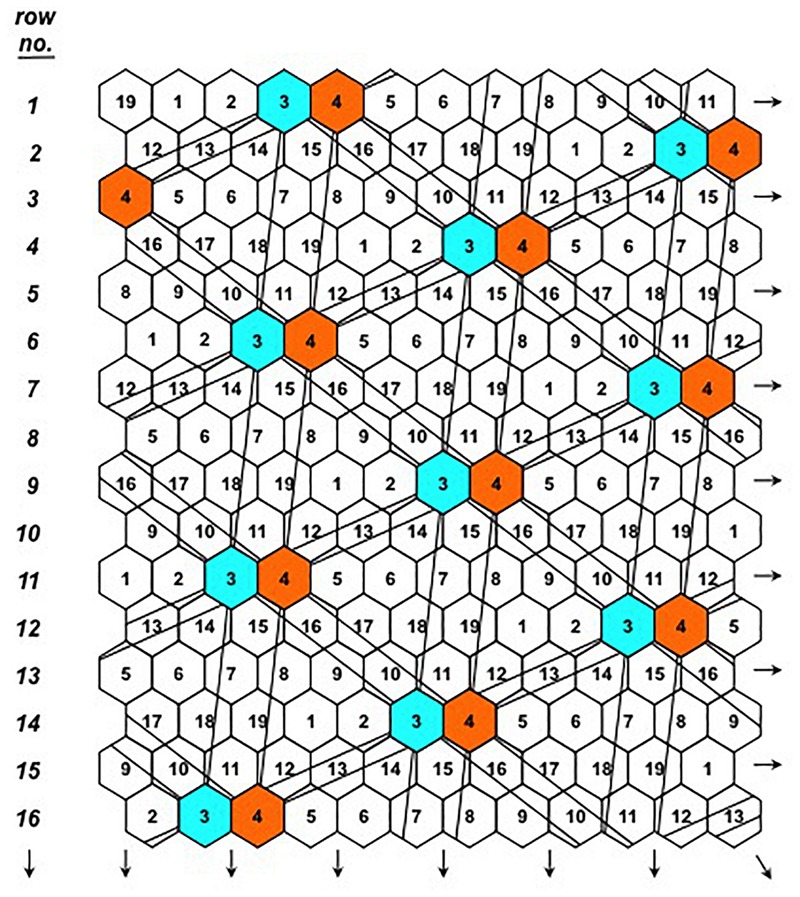
The D19 Honeycomb selection design for the evaluation of 19 different entries with design codes 1–19. The principle of the moving complete grids spreading across the selection field is illustrated for entries 3 and 4 and is valid for all design codes.

The first component of the equation is the plant yield potential, measured by the plant yield index $\text{pYI}={\left(x/\bar{x_{\text{r}}}\right)}^{2}$ which is unique and specific for each individual plant through the special arrangement of the Honeycomb selection designs (Figure 2). Each plant in every field position occupies the center of a complete and moving replicate and the yield value *x* of each central plant is expressed as a fraction with the denominator $\bar{x_{\text{r}}}$ being the average yield of all plants that form the moving replicate (Figures 2, 3). The second component of the equation is the plant stability of performance, measured by the coefficient of homeostasis or $\text{SI}={\left(\bar{x}/s\right)}^{2}$, where $\bar{x}$ and *s* are the mean plant yield and standard deviation, respectively, of the sibling line to which each plant belongs. Sibling plants of each line are allocated in a moving triangular grid (Figures 2, 4) spreading symmetrically across the field, thus $\bar{x}$ is the mean plant yield of each moving grid.

Further, the sibling line phenotyping or prognostic equation (sPE) was used to evaluate the sibling lines in each trial.

(2) $$\text{sPE}={\left(\bar{x}/s\right)}^{2}.{\left(x/\bar{x_{r}}\right)}^{2^{*}}$$

where the first parameter is the sibling line’s SI, as in pPE, and the second parameter is the mean plant yield index of all plants belonging to this sibling line. In addition to the use of moving complete replicates, the action of the two equations in reducing the masking effect of soil heterogeneity on single-plant yields is further enhanced by the moving triangular grids (Figures 2, 4) spreading symmetrically across the field to ensure effective sampling of soil heterogeneity and select for stability.

As the trials were progressing over the years, the individual plants were also phenotyped for their fodder and root biomass. During certain years and in order to cover as many variations as possible of those encountered in the farmers’ fields, the studies included some adjustments in the amount of water and fertilizer each plant received, as well as the use of plastic ground cover in the field (Figure 1A), a common practice among many local farmers in order to conserve water. Details of the yearly trials are provided below.

### Details of the 2009–2017 Trials

Year 1, 2009: A total of 315 cowpea plants were transplanted to the field on 14/05/2009 according to the layout of the D0 Honeycomb selection design that handles entries with no replications, since each plant of the 315 is considered to represent a unique entity. Twelve plants were selected and advanced to the next year’s trials based on their superiority regarding the pPE index of fresh pod yield after a total of five harvests. Each plant received a total of 72 L of irrigation water and 95 g of fertilizer (20-20-20), corresponding to 19 g N, 15.71 g K, and 8.21 g P.

Year 2, 2010: Seedlings of the 12 selected cowpea plants of the previous year were transplanted to the field on 09/06/2010 in two sub-trials, named A and B, according to the layout of two D7 Honeycomb selection designs (Figure 2) each capable to evaluate seven entries. Code numbers 1–6 in both sub-trials were occupied by the selected entries, while codes with number 7 by seed of the original landrace as a control. All codes comprised 36 individual plant replications for a total of 504 plants (7 × 2 × 36). Thirteen plants were advanced for the next year’s trials based on their superiority concerning the pPE index of fresh pod yield after a total of five harvests. Each plant received 78 L of irrigation water and 33 g of fertilizer (19-19-19) corresponding to 6.27 g N, 5.21 g K, and 2.69 g P. For the purposes of the present paper, only the sub-trial A is considered, since none of the lines included in the sub-trial B managed to out-yield others so as to reach the comparative trials of the final years (Supplementary Table S1).

Year 3, 2011: Seedlings of the 13 selected cowpea plants of the previous year were transplanted to the field on 19/05/2011 in two sub-trials, A and B, according to the layout of two D7 Honeycomb selection designs. Code numbers 1–7 in both sub-trials were occupied by the selected entries, with 45 replications of individual plants for all codes. Total number of plants in the trials was 630 and 13 were advanced in the next year based on their pPE index of fresh pod yield after a total of four harvests. Each plant received throughout the growing season 82 L of irrigation water and 42 g of fertilizer (19-19-19) corresponding to 7.98 g N, 6.62 g K, and 3.43 g P. For the purposes of the present paper, only the sub-trial A is considered, since none of the lines included in the sub-trial B managed to out-yield others so as to reach the comparative trials of the final years (Supplementary Table S1).

Year 4, 2012: Seedlings of the 13 selected cowpea plants of the previous year, along with seedlings of five plants of the 2010 trials that were deemed worth to be further advanced, were transplanted to the field according to the layout of the D19 Honeycomb selection design (Figures 3, 4) on 02/05/2012. Code numbers 2–18 were occupied by selected plants, while code number 1 was occupied by the control, with 30 replications of individual plants for all codes. Plastic cover was used across the whole field in order to study the effect of this practice on conserving water and the selection process. Total number of plants in the trial was 570 and 18 were advanced in the next year’s trials based on their pPE index of fresh pod yield after a total of seven harvests. Each plant received 46 L of irrigation water and 62 g of fertilizer (19-19-19) corresponding to 11.78 g N, 9.77 g K, and 5.06 g P. For the first time this year and throughout the remaining years, data from all individual plants included also the weight of the fodder biomass, in addition to the fresh pod weight.

Year 5, 2013: Seedlings of the 18 selected cowpea plants of the previous year were transplanted to the field according to the layout of the D19 Honeycomb selection design on 19/06/2013. Code numbers 1–18 were occupied by the selected plants, while code number 19 was occupied by the control, with 30 individual plant replications for all codes. Total number of plants in the trial was 570 and 18 were advanced in the next year’s trials based on their pPE index of fresh pod yield after a total of seven harvests. During this year, the Honeycomb trial was divided in three treatments, called “1,” “2,” and “3,” without affecting the plant arrangement in the field. In Treatment “1,” one-third of the plants, i.e., 190 plants, were placed under plastic cover receiving 43 L and 87 g of water and fertilizer (19-19-19) per plant corresponding to 16.53 g N, 13.71 g K, and 7.1 g P. The remaining two-thirds, i.e., Treatments 2 and 3, were placed in regular soil without cover, receiving 64 L of water per plant throughout the growing season. In Treatment “2,” plants received 45 g of fertilizer (19-19-19) corresponding to 8.55 g N, 7.09 g K, and 3.67 g P while plants grown under Treatment “3” received 34 g/plant of fertilizer by fertigation corresponding to 6.46 g N, 5.36 g K, and 2.77 g P throughout the growing season. For the first time this year and throughout the remaining years, the experimental handling of all plants included root biomass weighing after the last pod harvest. Thus, the dataset for each individual plant comprise its pod, fodder, and root weight.

Year 6, 2014: Seedlings of the 18 selected cowpea plants of the previous year were transplanted to the field according to the layout of the D19 Honeycomb selection design on 28/05/2014. Code numbers 1–18 were occupied by plants of the selected entries, while code number 19 was occupied by the control, with 30 replications for all codes. Total number of plants in the trial was 570 and 8 plants were advanced in the next year’s trials based on their pPE index values of fresh pod yield after a total of six harvests. Similar treatment arrangement to that of the 2013 Honeycomb trial was established. In detail, in Treatment “1” one-third of the plants, i.e., 190 plants, were placed under plastic cover receiving 24 L and 85 g of water and fertilizer (19-19-19) per plant corresponding to 16.15 g N, 13.40 g K, and 6.94 g P. The remaining two-thirds were placed in regular soil without cover, receiving 32 L of water per plant. In Treatment “2,” plants received 85 g of fertilizer (19-19-19) by fertigation, while plants grown under Treatment “3” received 16 g per plant of fertilizer by fertigation (19-19-19) corresponding to 3.04 g N, 2.52 g K, and 1.31 g P throughout the growing season.

Year 7, 2015: Seedlings of the eight selected cowpea plants of the previous year were transplanted to the field according to the layout of the D9 Honeycomb selection design on 27/05/2015. Code numbers 1–8 were occupied by the selected plants, while code number 9 was occupied by the control, with 21 replications for all codes. Total number of plants in the trial was 207 and 8 plants were advanced in the next year’s trials based on their pPE index of fresh pod yield after a total of seven harvests. During this year, the Honeycomb trial was divided in two treatments, called “1” and “2.” One half of the plants, i.e., 190 plants, were placed under plastic cover receiving 35 L of water and 90 g of fertilizer (19-19-19) per plant throughout the growing season corresponding to 17.10 g N, 14.93 g K, and 7.35 g P. In Treatment “2” plants were grown in uncovered soil, receiving 35 L of water and 90 g of fertilizer (19-19-19) per plant by fertigation corresponding to 17.10 g N, 14.93 g K, and 7.35 g P.

Year 8, 2016: Seedlings of the eight selected cowpea plants of the previous year were transplanted to the field according to the layout of the D9 Honeycomb selection design on 10/05/2016. Code numbers 1–8 were occupied by selected plants, while code number 9 was occupied by the control, with 40 replications for all codes. Total number of plants in the trial was 360 and 2 plants were advanced in the next year’s trial based on their pPE values. All plants were grown in regular soil without cover, receiving 70 L of water and 36 g of fertilizer (19-19-19) per plant in total during the growing period corresponding to 6.84 g N, 5.67 g K, and 2.94 g P.

Year 9, 2017: Seedlings of the two selected plants along with those of a single superior plant of the control were grown in the field following the arrangement of the D3 Honeycomb selection design on 11/05/2017. Ground cover was used and each plant received 30 L of irrigation water and 52 g fertilizer throughout the growing season corresponding to 9.88 g N, 8.2 g K, and 4.24 g P. Total number of plants were 234, corresponding to 78 single plant replications per code.

### Whole-Plant Phenotyping for Fodder and Root Biomass

As trials were progressing over the years, the individual plants were also phenotyped for their fodder (years 2012–2017) and root biomass (years 2013–2017) in order to study the lateral, indirect impact of the direct fresh pod selection process on those plant parts. After the final pod harvest and having secured at least few dry seeds of all individual plants to be used in further trials, the remaining above ground biomass (fodder) of each individual plant was cut and weighted, while at the same time all individual roots were excavated, thoroughly rinsed, and weighted on the spot.

### Evaluation of Selected Materials Under Dense Stands in Year 7 (2015)

The local “Argaka” landrace is valued for its fresh, long pods with harvesting performed by hand. In the farmer’s field, manual harvesting necessitates the adoption of wide distances among rows to allow the free movement of personnel. Thus, it is common among farmers to maintain a 2 m row-to-row distance, while adjusting the within row planting density, using 2 or even more seedlings around each drip of the irrigation tubing. To test the fresh pod yield performance of our selections at the commercial density used by farmers, a randomized complete block design was employed during 2015 to evaluate three superior entries selected during 2014, i.e., the families with design codes 7, 8, and 15, against the control. Bulk seed of the three entries and of the original seed source (control) was used in plots with four replications. Each plot consisted of a 22-m long row with 2 m distances between rows and two seedlings positioned around each drip every 60 cm.

### Statistical Analysis

During all years, selection of superior plants was based on the unique and specific value of the plant prognostic equation corresponding to each individual plant using appropriate JMP scripts (Fasoula et al., 2019). Selection pressures were generally maintained at ultra-high levels, starting from a 3.8% in the first year of selection and increasing up to 1.3% in subsequent years. Where appropriate, pairwise comparisons between means of different genotypes were performed using Student’s *t*-test at a significance level *p* < 0.05. Analysis of variance (ANOVA) was performed only during the densely grown trials of 2015. Regression analysis was used to evaluate the rate of pod yield increase of selected families during time. These analyses were performed using the RStudio environment (R Studio Team, 2016).

## Results

### Progress of Selection Through Whole-Plant Phenotyping of Fresh Pod Yield

**FIGURE 5:**
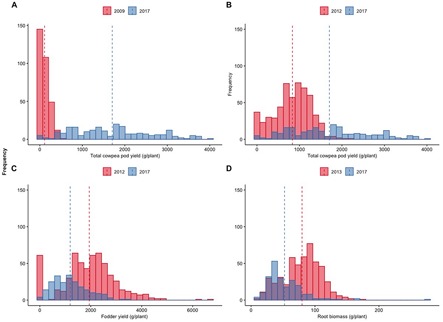
Distributions of total cowpea pod yield (g/plant) during the 2009 and 2017 growing seasons **(A)**, cowpea pod yield (g/plant) during the 2012 and 2017 growing seasons **(B)**, total fodder yield (g/plant) during the 2012 and 2017 growing seasons **(C)**, and root biomass (g/plant) during the 2013 and 2017 growing seasons.

The substantial progress achieved during the 9 years of selection for higher fresh pod yield in the local cowpea landrace “Argaka” is reflected in the change of frequency distributions of pod yields of individual plants between the first (2009) and the last year (2017) of the trials (Figure 5A). The negatively skewed distribution of 2009 with a maximum of 505 g pod weight of a single plant evolved to a completely different distribution with multiple novel yield classes and a maximum of 3,963 g pod weight of a single plant. The gradual transition of the distribution is evident in Figure 5B, when the intermediate selection year 2012 is considered against the last year.

The evolution between the first and last years of observations of the distribution of fodder and root biomass for which no direct selection was applied is demonstrated in Figures 5C,D, respectively. The movement of these distributions toward negative skewness highlights the indirect adaptive response of the plants directly selected for higher pod yields towards reduced fodder and root biomass.

Table 1 presents a summary of the analysis based on the properties of the Honeycomb selection designs and the sibling phenotyping equation (sPE) concerning the three best families selected each year versus the control for all years of selection. The data are also expressed in % of the best family in each year. The design code numbers in Table 1 do not represent the same lines across the different years, i.e., the line with design code 1 during 2011 is not derived from the line with code 1 during 2010, and so on. The highlighted lines in each particular year represent those that led to the best selection with design code 2 in the final year.

**TABLE 1:** Comparative values of the mean pod yield, the stability index (SI), and the sibling phenotyping equation (sPE) for three of the selected lines each year, signified by their corresponding Honeycomb design code numbers, along with the corresponding control during the years of selection.

**Year – design**	**Genotypes^1^**	**Mean pod yield (g)**	**% of best**	**SI**	**% of best**	**sPE**	**% of best**
2010 – D7	2^*^	398	100	7.99	100	11.6	100
	1	382	96	6.43	80	8.6	74
	3	344	86	7.53	94	8.1	70
	Control	276	69	4.57	57	3.18	27
2011 – D7	1	356	95	12.7	100	14.7	100
	5^*^	375	100	8.11	64	10.4	71
	2	350	93	8.4	66	9.4	64
	Control	Missing data					
2012 – D19	17	999	97	7.65	91	10	95
	14	977	95	8.45	100	10.7	100
	11^*^	970	94	6.58	78	8.17	77
	Control	596	58	2.46	29	1.15	11
2013 – D19	3	656	100	1.63	100	2.43	74
	8^*^	591	90	1.8	57	2.18	67
	7	590	90	2.71	86	3.26	100
	Control	538	82	1.89	60	1.9	58
2014 – D19	15	651	100	7.79	95	9.57	100
	8	646	99	6.46	79	7.83	82
	7^*^	637	98	8	98	9.43	98
	Control	492	75	2.9	35	2	21
2015 –D9	6	1,746	100	7.13	97	9.68	100
	5	1,561	89	7.24	98	7.85	81
	3^*^	1,543	88	7.37	100	7.81	81
	Control	1,460	83	3.87	53	3.67	38
2016 – D9	7	926	100	4	65	6.53	71
	4^*^	884	95	6.21	100	9.16	100
	5	788	85	5.47	88	6.4	70
	Control	495	53	1.71	27	0.79	9
2017 – D3	2^*^	1,690	100	3.6	99	3.78	100
	3	1,638	97	2.9	79	2.83	75
	1	1,613	95	3.6	100	3.5	92

*The values are also depicted in % of the highest line for this particular year. The design code associated with each year denotes the total number of entries evaluated during the year. The line codes with asterisks denote the pedigree of line with code number 2 which demonstrated the highest sPE value during the final year.*

**FIGURE 6:**
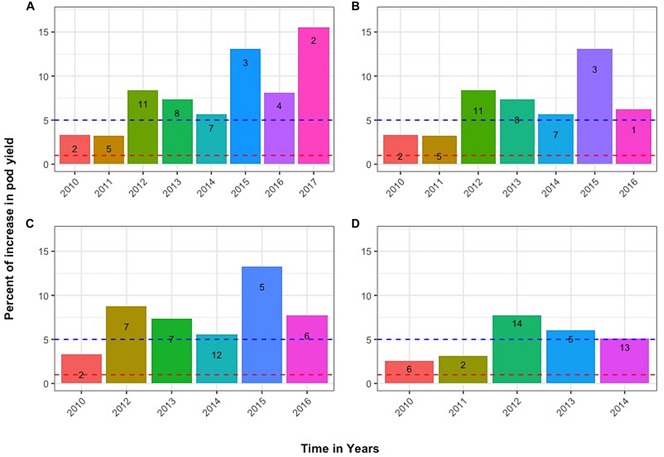
Tracing the advancement of selected sibling lines through the years. Numbers within bars represent the design code of each line in the corresponding year. Panels **(A,B)** depict the parallel fate of sibling plants derived from exactly the same lines until 2015 but diverged in 2016. Panel **(C)** shows the selection fate of another individual derived from the same mother plant in 2010. Panel **(D)** follows the fate of an individual derived from a different line in 2010 that failed to continue after 2014. Additional information provided in the text.

The progress across years of the individual progeny plants that led to the best line with design code 2 in 2017 is also presented in Figure 6A, using the respective data of total pod yield in grams. The histograms depict the % increase relative to the average control yield of the first trial year 2009 (level 0, corresponding to 112 g). Figure 6B depicts the parallel path and fate of another sibling plant that was derived from exactly the same sibling lines as the one in Figure 6A until the seventh trial year (2015), but diverged during 2016 becoming line with design code 1 and whose inferior performance based on the lower SI index and sPE values compared to line with design code 4 in Figure 6A prevented its inclusion in the 2017 final trial. This differentiation potential among sibling plants was observed throughout the years with all entries. Additional such cases are exemplified in Figures 6C,D. Figure 6C follows the selection fate of another individual plant derived from the same mother plant in 2010 whose progenies failed to continue after 2016, while Figure 6D follows the fate of a plant originating from a different mother plant in 2010, that failed to continue after selection year 2014. Notably, many lines with transiently higher pod yields in grams, but with lower SI index values, were not able to sustain their superiority through the environments and years of selection. Consequently, they were not advanced in the final trials.

The summary statistics of the trials for each year are presented in Supplementary Table S2. The data are the collective output of the total pod yield of the year and do not discriminate among individual entries. The rate of pod yield increase across years is derived as customary (Falconer and Mackay, 1996; Tefera et al., 2009) from the slope of the linear regression of pod yields of the selections on the corresponding year of the trials, representing the average rate of increase per year. It is at the level of 180 g per plant per year, highly significant (*p* < 0.001) with *R*^2^ = 0.64 (Supplementary Figure S1, depicting all the individual plant replications of the selected families, as well as the corresponding frequency distributions of each year).

The relative genetic gain can be estimated from the slope of the linear regression (Tefera et al., 2009) as the ratio of increase to the corresponding mean values of the control and expressed as a percentage. Considering that the mean value of all the individual control plants across years and environments is 655.3 g per plant, the relative annual genetic gain is estimated at 27.46%. This result is also in agreement with the 31.37% superiority of the best selected family vs. the control in the densely grown trials (Figure 12).

### Whole-Plant Phenotyping for Fodder and Root Biomass

**FIGURE 7:**
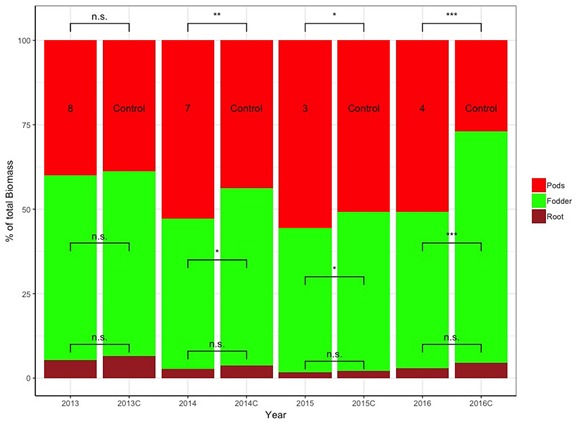
Stacked bar graph of the relative differences in % of the total plant biomass comprising pods, fodder, and roots between the control and selected lines with the respective design codes presented within each histogram for years 2013–2016. Asterisks denote significant differences between the selected genotypes and the control within the same year by paired *t*-test for each plant part (pod, fodder, and root biomass). NS, not significant; ^*^p < 0.05; ^∗∗^*p* < 0.01.

Figure 7 depicts the relative differences in % of the total plant biomass comprising pods, fodder, and roots between the control and selected lines with the respective design codes presented within each histogram for years 2013–2016. The actual values in grams corresponding to 100% are the following: for 2013, 1,476 and 1,387 g for family 8 and the control, respectively; for 2014, 1,204 and 1,123 g for family 7 and the control, respectively; for 2015, 2,644 and 2,875 g for family 3 and the control, respectively; and for 2016, 1,847 and 1,887 g for family 4 and control, respectively.

The results in Table 2 demonstrate the big differences in sPE values for pod, fodder, and root biomass among families selected for their pod yield. For example, in 2016, family 4 has the highest sPE values for pod yield and fodder biomass, but one of the lowest regarding root biomass. These results are complementary to Figures 9–11.

**TABLE 2:** Comparative values of the sPE for pod, fodder, and root biomass among the selected lines of Table 1 during the years of selection.

**Year – design**	**Genotypes**	**sPE for pod yield**	**% of best**	**sPE for fodder biomass**	**% of best**	**sPE for root biomass**	**% of best**
2012 – D19	17	10	95	4.37	21	No root data taken in 2012	
	14	10.7	100	4.30	20		
	11^*^	8.17	77	10.30	49		
	Control	1.15	11	8.80	42		
2013 – D19	3	2.43	74	2.8	56	5.6	52
	8^*^	2.18	67	3	60	5.9	55
	7	3.26	100	4	79	10	95
	Control	1.9	58	1.7	33	9.8	91
2014 – D19	15	9.57	100	6.6	51	11	60
	8	7.83	82	6.1	47	10.8	58
	7^*^	9.43	98	8.7	67	12.7	68
	Control	2	21	13	100	8.9	48
2015 – D19	6	9.68	100	13	96	15	74
	5	7.85	81	7	51	7.8	38
	3^*^	7.81	81	8.4	62	11	54
	Control	3.67	38	9	66	21	100
2016 – D19	7	6.53	71	6.2	98	13.6	100
	4^*^	9.16	100	6.3	100	5.3	39
	5	6.4	70	3.9	62	6.2	46
	Control	0.79	9	5.4	87	12.6	93
2017 – D19	2^*^	3.78	100	3.25	97	5	100
	3	2.83	75	3.4	100	4.4	87
	1	3.5	92	3.22	96	1.9	38

*The values are also depicted in % of the highest line for this particular year. Notably, the fodder and root sPE values do not necessarily contain the highest line with the 100% value of the particular trial, as the direct selection was only for high pod sPE. The line codes with asterisks denote the pedigree of line with code number 2 which demonstrated the highest pod sPE value during the final year.*

### Ground Cover and Fertigation Treatments

During the years 2013–2015 the trials included treatments comprising the use or not of plastic ground cover, as well as differential fertigation regimes described in the section “Materials and Methods.” These artificially created microenvironments within the selection field were used to derive a comprehensive SI for each family incorporating all responses of the individual plants across the field. This is possible since the response of each plant is not affected by its neighbors due to the wide distances employed. The multiple replicates, ranging from 30 to 78 across years, permit accurate selection in all fertility gradients. This is further supported by the *t*-test comparisons between the best family and the control within each year of selection (Supplementary Figure S2). During the final year 2017, the best family, selected under the various microenvironments described, exhibited a statistically significant difference compared to the control. This acquires additional importance, since the control of 2017 was derived from the single best control plant of 2016 instead of the bulk control seed.

**FIGURE 8:**
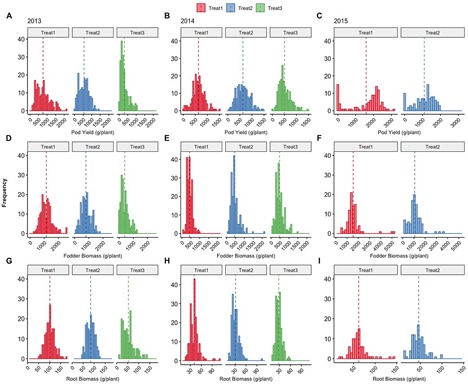
Distributions of total pod yield **(A–C)**, fodder **(D–F)**, and root biomass **(G–I)** of cowpea plants (g/plant) grown under different treatments (Treatments 1, 2, and 3) across years 2013, 2014, and 2015. Dashed vertical lines denote the mean value of each parameter, details in the text.

Figure 8 shows the comprehensive picture of the individual plant frequency distributions across treatments and years. Treatment 1 consists of the use of ground cover with reduced fertigation, whereas Treatments 2 and 3 have no cover, with Treatment 3 receiving the least fertigation. The more stressful environment represented by Treatment 3 within years 2013 and 2014 is consistently reflected in the negatively skewed distributions of pod yield for which direct selection was applied and to a lesser degree in the fodder and root biomass for which there was no direct selection. When examining the trends across years for pod yield, there is a clear trend toward positive skewness of the relevant distributions, indicating that selection is effective regardless of the starting year or imposed treatments. Within 2015, the differences in the frequency distribution concern only the use or not of plastic ground cover with the same fertigation between treatments.

**FIGURE 9:**
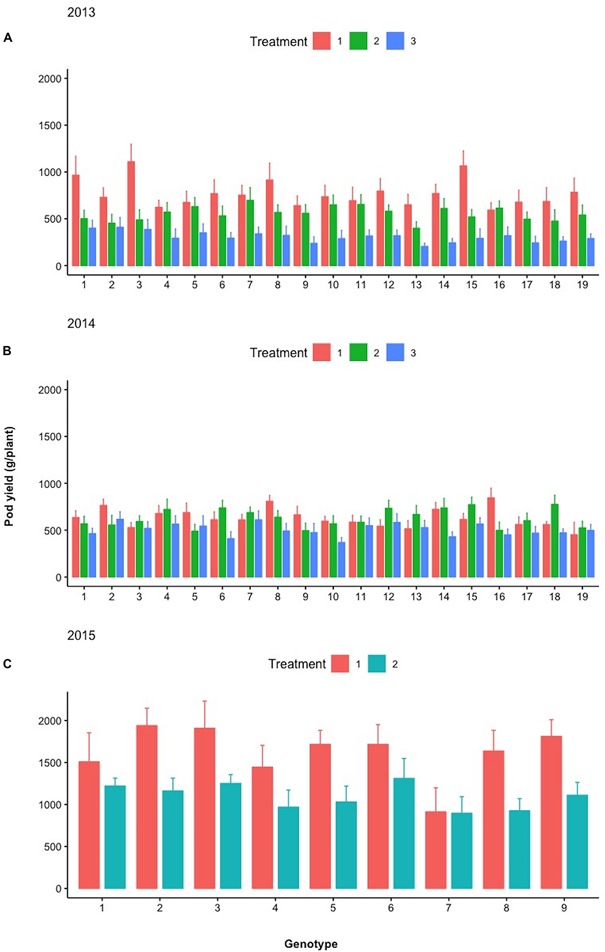
Mean pod yield (g/plant) of the different lines examined during **(A)** 2013, **(B)** 2014, and **(C)** 2015 under the different treatments (Treatments 1, 2, and 3). Spreads in the bar plots denote the standard error of the mean of each line.

Figure 9 depicts the response of each sibling family or line to the differential treatments within each year for pod, fodder, and root biomass. During years 2013 and 2014, 19 families were evaluated in a D19 Honeycomb selection design, whereas in year 2015, 9 families were evaluated in a D9 design. A striking observation is the fact that there are sibling families that are less affected by the differential treatments, as for example, families 3 and 11 in 2014 and family 7 in 2015. The various degrees of stability across treatments demonstrated by sibling lines, i.e., derived from the same mother plant, are notable. Again, pod biomass production is higher in Treatment 1 (ground cover) and the same is true for root biomass production across years. The latter was not necessarily anticipated since the response of root biomass to ground cover has not been previously documented.

### Performance Under Commercial Densities

During the seventh year of the trials (2015), a parallel comparative trial was performed in order to evaluate the families selected based on their higher sPE with the control under the commercial planting densities used by local farmers. The results are depicted in Figure 12. All three sPE-selected families have significantly higher total pod yields compared to the control with the highest differences amounting to 31.37%. Further, they have significantly higher pod yields during certain of the individual and consecutive harvests with most notable and of high relevance the significant differences in the first two harvests that define the earliness of the variety. Interestingly, the control appears superior during the 3rd, 4th, and the last harvest, but this temporal superiority is not sustained when the overall pod yield is considered taking into account all consecutive harvests.

## Discussion

In conventional breeding schemes the entries under consideration for selection are traditionally evaluated in as uniform soil and environmental conditions as possible in order to extract reliable information about potential superiority. Otherwise, the Genotype × Environment (G×E) interactions interfere and minimize progress through selection. This standard knowledge about uniformity requirements is not of concern when evaluation is performed at the individual plant level as described in this series of trials. The properties of the Honeycomb selection designs permit the identification of superior plants in all fertility and moisture gradients within the selection field (Figures 2–4). Further, in all years after year 1 when the initial farmer germplasm was evaluated, the entries consisted of sibling lines derived from a small number of corresponding mother plants with the majority of them derived from a single, superior mother plant identified during the 2010 evaluation (Supplementary Table S1). Comparison among sibling lines offers significant advantages, including the possibility to evaluate plants concurrently on the basis of the two components of the crop yield potential expressed as unique individual values of the plant phenotyping equation assigned to each single plant, and the possibility to predict performance in the same generation of selection without the need to await results from the progeny testing in subsequent years (Fasoula, 2006, 2008, 2013; Greveniotis and Fasoula, 2016).

The partitioning of crop yield potential into components that can be reliably evaluated at the single plant level under the conditions ensured by the Honeycomb selection designs (Fasoula and Fasoula, 2000, Fasoula and Fasoula, 2002, 2018a) is a major step in improving progress through selection, especially considering the early segregating generations of a breeding program as well as the landrace-type of materials, where each seed has a unique genetic profile and there is not enough seed for conventional replications. This lack of adequate seed supplies is the main reason behind the very common practice of visual selection until enough seed is secured to allow replications, commonly between 3 and 6, of the densely grown plots. The obvious result of this postponement in evaluation is the irretrievable loss of potentially valuable genotypes. In the Honeycomb trials the number of replicates is commonly 30–100 and can easily increase as necessary since the unit of evaluation and selection is the individual plant grown in the absence of any interplant interference and not the densely grown plot. The relevance of the above in the present work is the results in Figure 12, where the comparison between the control original material and the selected lines is performed under the usual commercial densities. Figure 12 is also discussed below.

The results in Figure 5A highlight the novel classes of yield variations that appeared in the last year of direct selection for fresh pod yield compared to the original control material and the tremendous increase in yield, where the highest yielding individual plant during 2017 had a fresh pod yield of 3,963 g, i.e., almost 4 kg. Of interest are also the results in Figures 5C,D showing the adaptive responses in fodder and root biomass demonstrated by the selected families through the years, in order to be able to sustain the increased pod yield for which direct selection was applied at ultra-high selection pressures (percentage of selected individual plants each year ranges from 3.8 to 1.3). The progress to selection or genetic gain in a breeding program is directly proportional to the selection intensity (Falconer and Mackay, 1996), and the higher the selection intensity, i.e., the less plants advanced in the next generation of selection, the greater the progress. The average selection pressure in a conventional breeding program is commonly around 10% so as to avoid loosing potentially valuable materials due to imprecise evaluation (e.g., lack of enough seed for replications, visual evaluation). To estimate the rate of genetic gain in plant breeding, there exist various approaches, commonly using the linear regression of yield on different time points of the period under consideration comparing the new selections to the controls, where the slope of the line corresponds to the average increase per year (Tefera et al., 2009). In the current series of trials, the average rate of increase in pod yield per year is 180 g per plant (Supplementary Figure S1).

The results in Table 1 demonstrate that lines with the highest pod yield values are not necessarily those with the highest sPE values, as the SI can greatly differentiate the outcome. This is particularly evident when considering the control that has consistently lower SI and equation values since it has not been selected for stability. For example, during 2015, the control has a much lower sPE value due to decreased stability, although it has the highest mean pod yield compared to other years.

For a more direct perception of the progress through selection, the histograms in Figure 6 are presented, derived from the actual yields in grams of each sibling family across years, and not from the corresponding pPE values. The pPEs of each family represent unitless values that take into consideration the yields in grams within each moving replicate (Figures 2–4) and the SI of each sibling family based on the performance of family members within their own triangular grid extending across all field gradients. Figure 6 shows how sibling plants derived from the same mother plant and belonging to the same sibling line for a number of selection years can greatly diverge through the years and through exposure to the various, even if only slightly different, soil and environmental conditions encountered in this particular field. Cowpea is a highly self-fertilizing species with cross-pollination <2% (OECD, 2016) and this capacity for differentiation concerns both the higher yielding families and the lower yielding ones during all years in the trials. In no case a family with a low comparative sPE value produced progenies that gave rise during the next years to families with high comparative pPE values. The above testify against the occasional random cross-pollination effects. Further, the ultra-wide distances (2 × 1.8 m) employed in the trials are not conducive to cross-pollination by insects.

The ability for clear differentiation among sibling plants derived from seeds of the same mother plant whose superiority persists through the years highlights the great resolving power of the methodology toward uncovering the untapped genetic or even epigenetic potential among very close relatives. This opens the way to the possibility to derive closely related materials with differential adaptation to micro-environments within a single growing season and is particularly relevant when considering climate change effects and the need for quick adaptation to those (Singh et al., 2013). The results in Figures 5C,D are further elaborated in Figure 7 where the relevant proportion of pod, fodder, and root biomass is depicted for 2013–2016 for a series of selected families against the control. The progression of the selection process created a clear trend where the selected materials have a significantly higher proportion of pod compared to fodder biomass, and a consistently lower, although not significantly different, root biomass. It is noteworthy to mention that the ultra-wide distances employed in the Honeycomb designs are ideal to perform phenotyping studies of roots under real field conditions as the roots do not become entangled and the within-field variability is effectively controlled. Root phenotyping is a procedure fraught with difficulties and is rightly described as a phenotyping bottleneck (Fiorani and Schurr, 2013). Over the years, the comprehensive and labor-intensive process of phenotyping individual plants in terms of fodder and root biomass was performed in a total of 2,511 plants, corresponding to >5,000 weighing cases in total, whereas the pod phenotyping was performed in >4,000 plants in multiple harvests during each growing season, corresponding to >27,000 cases in total.

Importantly, since the direct selection process did not concern fodder and root biomass, their measurements reflect the indirect changes sustained by the plants selected for higher pod yields relative to the unselected control. During all years the same trend is observed, i.e., control plants have consistently lower pod and higher fodder and root biomass compared to the selections, pointing to the fact that the effects of indirect selection led to plants with differential allocation of resources within. Differences are statistically significant as indicated in Figure 7.

Cyprus cowpea farmers use a variety of irrigation and fertilization regimes to produce their crop. The common element in most cases is the use of drip irrigation in varying amounts. The use of plastic ground cover is known to conserve irrigation water and is preferred by many. However, it requires special equipment and not all farmers are using it. Further, not all farmers agree on the amount and mode of fertilization to be used, although it is recognized that the application of fertigation vs. ground fertilizer requires a more progressive mindset and some basic equipment.

In the trials reported here, we experimented with a variety of combinations, as described under the individual years. This is another important and flexible option allowed by the Honeycomb selection designs, since the consumption of resources of each individual plant in its own micro-environment is not affected by its neighbors and can be precisely monitored and evaluated without the masking effects of competition and soil heterogeneity. These micro-environments are created because of the native field heterogeneity, as well as because of the artificially imposed ground cover and fertigation treatments. The collective performance of the micro-environments corresponding to each family line is used to derive the unitless SI, a comprehensive measure of stability of performance leading to the identification of the most stable entries. Further, the conditions in the Honeycomb trials allow the application of very precise nutrition and irrigation regimes on a per plant basis, creating conditions that exactly correspond to the ideal of precision agriculture. Thus, in our approach, the environmental differences are not to be avoided, but to be exploited in order to select for stability of performance across years and environments. In further testament to the success of selection for stability exploiting the different microenvironments, the comparisons of the results between years 2012 and 2017, where the ground cover was used across the whole field, are most interesting. In particular, during 2012, each plant received 46 L of irrigation water and 11.78, 9.77, and 5.06 g of N, P, and K nutrients, respectively, whereas in 2017 the corresponding values were only 30 L of irrigation water and 9.88, 8.20, and 4.24 g of N, P, and K nutrients per plant. This further testifies to the high rate of increase achieved and the corresponding high genetic gain.

**FIGURE 10:**
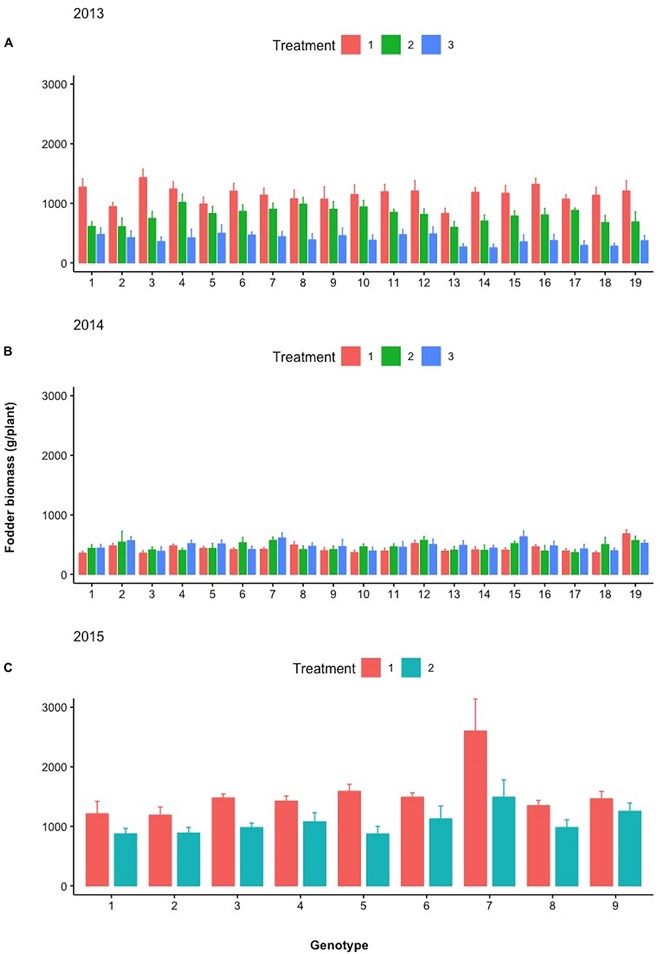
Mean fodder biomass (g/plant) of the different lines examined during **(A)** 2013, **(B)** 2014, and **(C)** 2015 under the different treatments (Treatments 1, 2, and 3). Spreads in the bar plots denote the standard error of the mean of each line.

**FIGURE 11:**
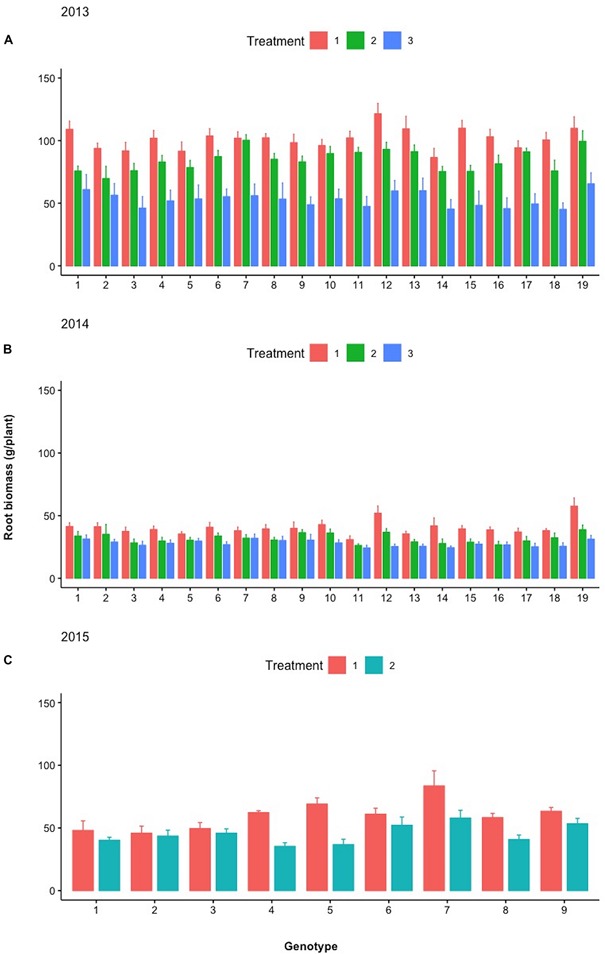
Mean root biomass (g/plant) of the different lines examined during **(A)** 2013, **(B)** 2014, and **(C)** 2015 under the different treatments (Treatments 1, 2, and 3). Spreads in the bar plots denote the standard error of the mean of each line.

Further, the use of ground cover demonstrated the capacity to increase pod yield and identify individual plants with increased ability to exploit resources reaching an extremely high pod yield potential (Figures 8–11 and Supplementary Figure S1). This is in agreement with the use of ground cover by the farmers to conserve water without sacrificing yield. The fact that we used both ground cover and uncovered soil in the selection process, along with differential fertigation treatments, led to the creation of lines that perform very satisfactorily in different environments covering a wide range of farmer practices. When examining the trends across years for pod yield in Figure 8, there is a clear trend toward positive skewness of the relevant distributions as in Figure 5, indicating that selection was effective regardless of the starting year or the imposed treatments.

During the different treatments in 2013–2015 (Figures 9–11), large crossover effects became evident (Table 2), where there are sibling lines performing very different under the contrasting regimes. In Figure 9, this is the case for the pod yield of the lines with design codes 15 and 16 in 2013 that are sibling lines derived from the same plant during 2012. Similarly, in 2014, the sibling lines with design codes 5 and 6 exhibit rather contrasting performance among treatments. In Figure 10, where the fodder biomass is depicted, the lines with design codes 17 and 18 in 2014 are sibling lines derived from the same plant of 2013. In the case of root biomass in Figure 11, analogous example is the differences in 2013 between sibling lines 6 and 7, which have been derived from the same mother plant in 2012. There are also cases where certain lines are differentially affected by the treatments in their pod yield vs. fodder and root biomass (2015 line with design code 7, Figures 9–11, where the treatments did not affect as much the pod yield). Our approach demonstrates that cross-over effects can be successfully exploited to identify lines with increased stability of performance.

The results in Table 2 further demonstrate the high discrimination potential of the sPE and the fact that lines with the highest sPE values for pod yield are not necessarily the same with those having the highest sPE values for fodder or root biomass. Root phenotyping is of paramount importance for agriculture, including low-input systems and marginal environments (Fiorani and Schurr, 2013). Breeding efforts that select for specific root traits are limited despite the importance of the root systems (Paez-Garcia et al., 2015). This series of trials describes a whole-plant root phenotyping method, readily transferable to other crops, concurrently connecting the results with the above ground biomass and commercial yield under real field conditions and under a variety of irrigation and fertilization regimes.

**FIGURE 12:**
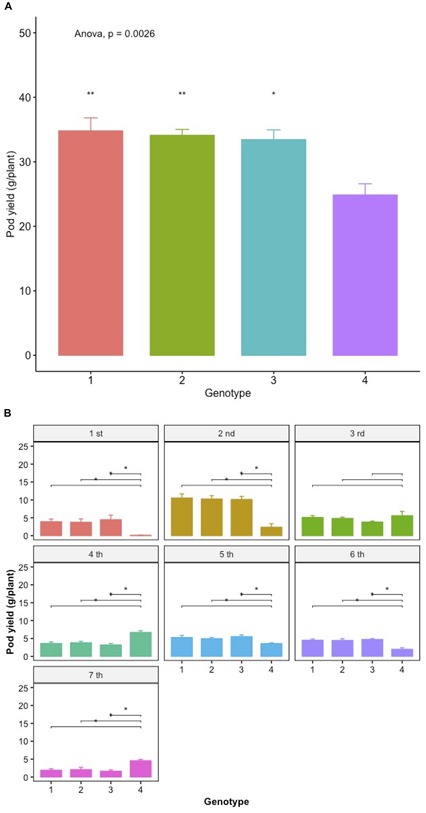
Mean pod yield (g/plant) of lines selected on the basis of their high spE values and the control under commercial planting densities **(A)** and the mean pod yield after each harvest event **(B)**. Spreads in the bar plots denote the standard error of the mean of each genotype. Asterisks denote significant differences between the selected line and the control by paired *t*-test. Lack of asterisk, not significant; ^*^p < 0.05; ^∗∗^*p* < 0.01.

The results in Figure 12 show the pod yield superiority of three lines from the selection process against the control both in terms of total pod yield and pod yield of the individual harvests under commercial planting densities. The selected lines have significantly higher total pod productivity with the difference from the control amounting to 31.37%. Further, they are more productive during certain of the consecutive harvests with most notable and of high relevance the differences in the first two harvests that define the earliness of the variety and the time that the first fresh pods of the year will reach the market commanding the highest prices. This fact is of great interest to farmers who commonly use low plastic tunnels to raise the soil temperature and protect the seedlings in early spring in order to be able to produce the early pods of the season. Thus, this management practice can be further enhanced by the genetic improvement toward earliness as evidenced in the superior selections. Notably, the accomplishment of earliness was another indirect consequence of the direct selection for yield, and not for secondary traits related to yield.

In addition to the direct applications towards increasing cowpea pod yields and enhancing farming practices, this long-term series of trials produced a range of valuable and contrasting genotypes, comprehensively characterized for quantitative traits at the whole-plant level that are available to the community for additional genomic studies. The overall cowpea genomic resources available today can further elucidate the underlying causes of our observations. A whole-genome shotgun (WGS) assembly was reported for cowpea (Muñoz-Amatriaín et al., 2017), while most recently, the whole cowpea genome was sequenced (Lonardi et al., 2019).

## Conclusion

In this work, we demonstrate a continuous progress to selection with high shifts in the progeny mean compared to the parental mean that starts from the first generation of selection. To ensure that this progress is continuous and reproducible, we used many years and environmental conditions, while including the original farmers seed as a control. In addition and of relevance to this project, cowpea pod yield is sensitive to the heat conditions encountered each year that can affect flowering and pod setting. In the particular Cyprus landrace that we work with, this critical period is encountered up to seven times per year, since the fresh pod harvest takes place continuously for up to 3 months. So, each selected plant in the trials has responded successfully to multiple environmental screenings and outyielded its siblings. And yet, this hard-earned superiority can sometimes be overcome during the next generation of selection. Owing to the precision of our approach, we demonstrate that sibling lines get continuously differentiated and despite the very similar genetic background and common origin from the same mother plant, not all of them are advanced with equal success during the 9 years of selection. This by itself is a very interesting outcome.

The series of trials described here demonstrate the great plasticity of the cowpea genome and an apparently inexhaustible potential for high response to selection under ultra-high selection pressures during a decade. The key to this impressive outcome is the focus of phenotyping and selection on the individual plant grown at field distances that exclude any interplant interference with the equal sharing of resources (i.e., there is no interplant competition) using innovative selection designs that precisely account for all aspects of the native field heterogeneity, as well as of the artificially imposed treatments. The analysis of crop yield potential to two components that can be accurately and precisely estimated at the level of the individual plant is a key element to the success of the process. Direct selection for yield was successfully implemented at all stages using no indirect selection for secondary traits related to yield.

The possibility to identify within a single growing period divergent sibling lines from seeds of the same mother plant in a highly selfing species and at any point during the long-term selection process, further testifies to the potential of the approach. These lines show quick differential adaptation to micro-environments, an important fact especially when considering climate change and the urgent need for efficient methods to respond to it. Thus, the new focus, apart from the simple variety maintenance should include their continuous improvement and exploitation of micro-adaptation processes for different fields. Last but not least, this work presents a novel approach to the multiple challenges of root phenotyping and a means to meaningfully associate it with whole-plant performance in the field. The precisely characterized individual plants in terms of their quantitative responses to contrasting environments will be useful to deeper investigate the notion of one individual whole-plant phenotype corresponding to one individual genome through the currently available cowpea genomic resources and will also serve to feed modeling approaches in the future.

## Author Contributions

DF conceived and planned the work. MO and DF equally contributed to the data analysis and writing of the manuscript. All authors discussed, reviewed, and approved the manuscript, and contributed to the trial execution.

## Conflict of Interest Statement

The authors declare that the research was conducted in the absence of any commercial or financial relationships that could be construed as a potential conflict of interest.

## Supplementary Material

FIGURE S1 Relationship between cowpea pod yield of individual plants of selected lines of the local landrace “Argaka” and year of selection. The corresponding pod yield frequency distributions are depicted below the regression graph. The slope of the linear regression represents the average rate of annual increase in pod yield (180 g/plant).

FIGURE S2 *T*-test comparisons between the best family and the control within each year.

TABLE S1 Tracking the progenies of selected sibling lines in the course of 9 years of selection as described under the section “Materials and Methods.” Notably, the final exceptional Line 2 in the last year of selection (2017) was derived from one exceptional plant of the original farmer’s seed in 2009 that gave Line A2 during 2010 in yellow. However, not all progenies of Line A2 were able to outperform other plants and lines and thus, they were not advanced with equal success during the subsequent years. This is also the case with the progenies of other lines that were formed during the years of selection highlighting the sensitivity of selection achieved when using the phenotyping equations.

TABLE S2 Summary statistics per year of all trials during the selection considering the total output of the trial, not the individual lines.
