# Supplementary figures and images for: Optimizing Resource Allocation in a Cowpea (Vigna unguiculata L. Walp.) Landrace Through Whole-Plant Field Phenotyping and Non-stop Selection to Sustain Increased Genetic Gain Across a Decade
Source: Front Plant Sci. 2019 Aug 7;10:949. doi: 10.3389/fpls.2019.00949 (PMC6694199; doi:10.3389/fpls.2019.00949)

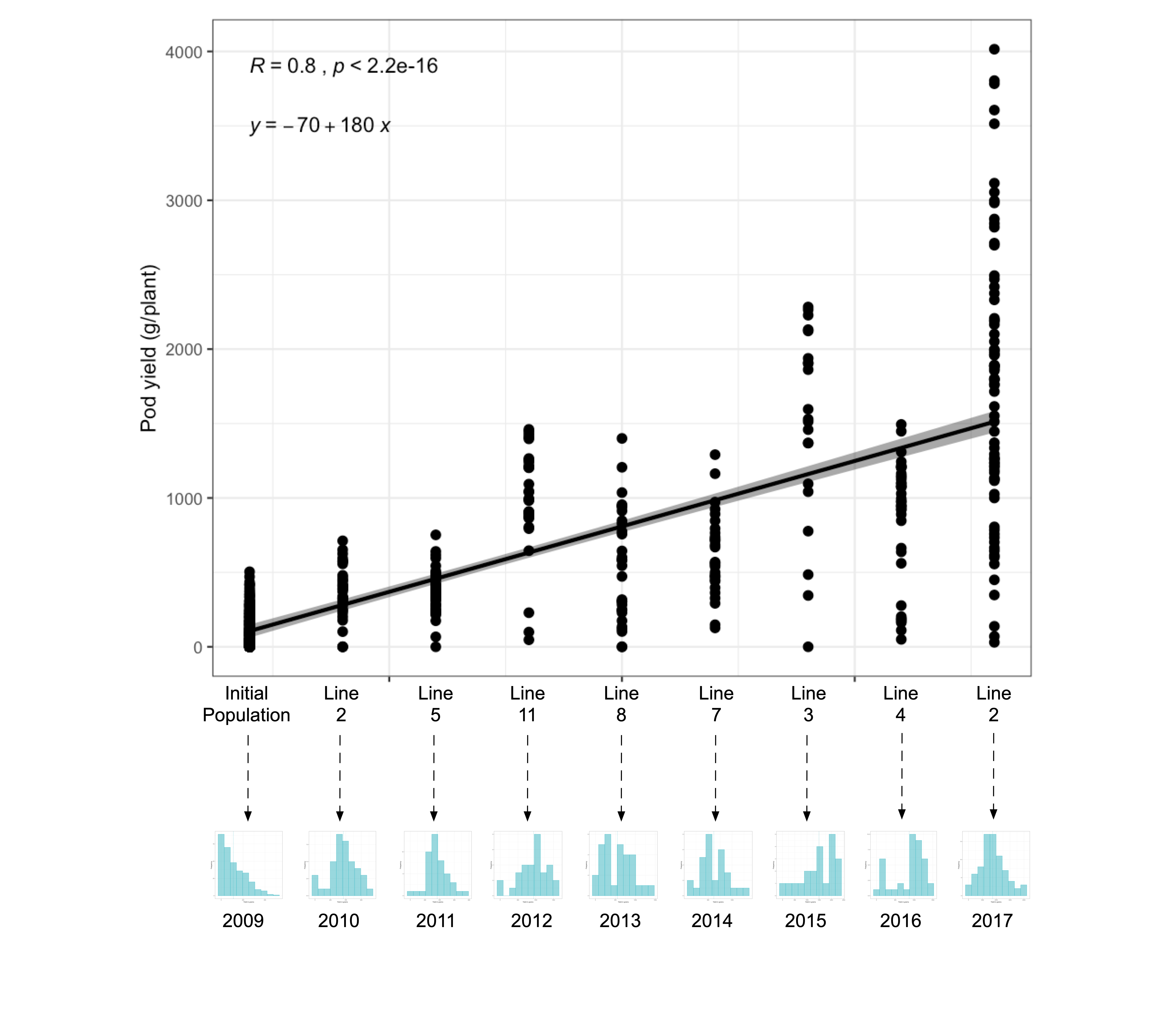

Supplement: FIGURE S1 — Relationship between cowpea pod yield of individual plants of selected lines of the local landrace “Argaka” and year of selection. The corresponding pod yield frequency distributions are depicted below the regression graph. The slope of the linear regression represents the average rate of annual increase in pod yield (180 g/plant). [file Image_1.tiff]

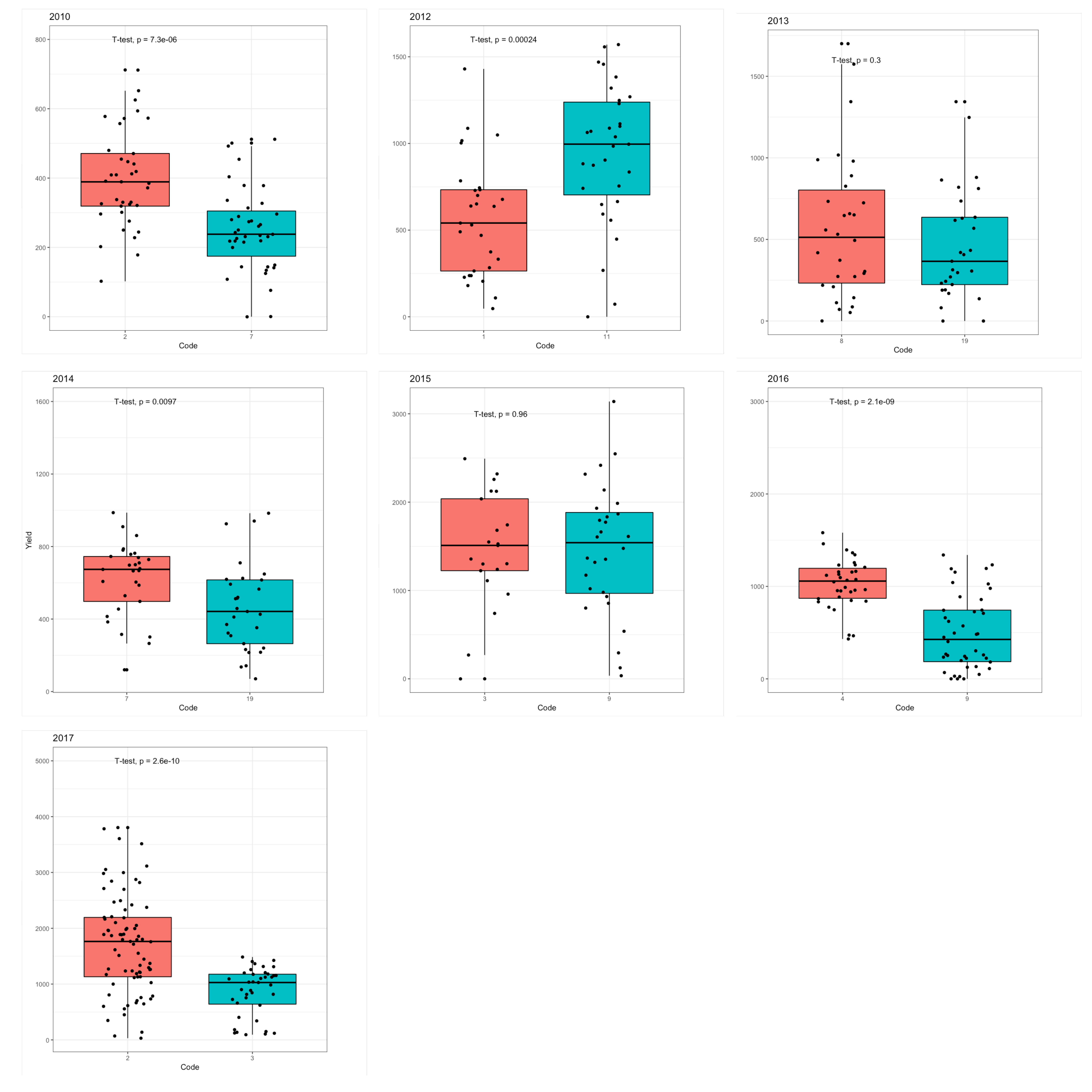

Supplement: FIGURE S2 — T-test comparisons between the best family and the control within each year. [file Image_2.TIFF]
