# Supplementary material for: Optimizing Resource Allocation in a Cowpea (Vigna unguiculata L. Walp.) Landrace Through Whole-Plant Field Phenotyping and Non-stop Selection to Sustain Increased Genetic Gain Across a Decade
Source: Front Plant Sci. 2019 Aug 7;10:949. doi: 10.3389/fpls.2019.00949 (PMC6694199; doi:10.3389/fpls.2019.00949)
Supplement: TABLE S1 — Tracking the progenies of selected sibling lines in the course of 9 years of selection as described under the section “Materials and Methods.” Notably, the final exceptional Line 2 in the last year of selection (2017) was derived from one exceptional plant of the original farmer’s seed in 2009 that gave Line A2 during 2010 in yellow. However, not all progenies of Line A2 were able to outperform other plants and lines and thus, they were not advanced with equal success during the subsequent years. This is also the case with the progenies of other lines that were formed during the years of selection highlighting the sensitivity of selection achieved when using the phenotyping equations. [file Table_1.DOCX]

Supplementary Table 1.

| SUB-TRIAL A | | | | | | | |
| --- | --- | --- | --- | --- | --- | --- | --- |
| **2010** | **2011** | **2012** | **2013** | **2014** | **2015** | **2016** | **2017** |
| A1 | A1 | 9 |  | | | | |
| A1 | A1 | 10 |  | | | | |
| A1 | A1 | 12 |  | | | | |
| A1 | A6 |  | | | | | |
| A2 |  | 4 |  | | | | |
| A2 |  | 5 | 11 | 2 | 8 |  | |
| A2 |  | 5 | 12 | 5 |  | | |
| A2 |  | 5 | 12 | 6 |  | | |
| A2 |  | 6 |  | | | | |
| A2 |  | 7 | 6 |  | | | |
| A2 |  | 7 | 7 | 4 | 2 |  | |
| A2 |  | 7 | 7 | 8 | 4 |  | |
| A2 |  | 7 | 7 | 12 | 5 | 6 |  |
| A2 |  | 8 |  | | | | |
| A2 | A5 | 11 | 8 | 7 | 3 | 1 |  |
| A2 | A5 | 11 | 8 | 7 | 3 | 4 | 2 |
| A2 | A5 | 11 | 9 |  | | | |
| A2 | A5 | 11 | 10 |  | | | |
| A2 | A5 | 13 |  | | | | |
| A2 | A7 |  | | | | | |
| A3 |  | | | | | | |
| A4 |  | | | | | | |
| A5 |  | | | | | | |
| A6 | A2 | 14 | 4 |  | | | |
| A6 | A2 | 14 | 5 | 13 |  | | |
| A6 | A3 |  | | | | | |
| A7 | A4 |  | | | | | |

Supplementary Table 1 (Continued)

| SUB-TRIAL B | | | | | | | |
| --- | --- | --- | --- | --- | --- | --- | --- |
| **2010** | **2011** | **2012** | **2013** | **2014** | **2015** | **2016** | **2017** |
| B1 | B4 |  | | | | | |
| B2 |  | | | | | | |
| B3 | B1 |  | | | | | |
| B3 | B7 |  | | | | | |
| B4 |  | | | | | | |
| B5 |  | | | | | | |
| B6 | B2 | 17 | 1 |  | | | |
| B6 | B2 | 17 | 2 | 9 |  | | |
| B6 | B2 | 17 | 2 | 10 |  | | |
| B6 | B2 | 17 | 2 | 11 |  | | |
| B6 | B2 | 17 | 3 | 3 | 1 |  | |
| B6 | B2 | 18 | 17 |  | | | |
| B6 | B2 | 19 |  | | | | |
| B7 | B5 |  | | | | | |
| B7 | B6 | 16 |  | | | | |

Supplementary Table 2.

| Year | Mean Yield  (g/plant) | Standard  Deviation | Coefficient of Variation (%) | Mean Yield per consecutive harvest  (g/plant) | Standard  Deviation | Coefficient of Variation (%) |
| --- | --- | --- | --- | --- | --- | --- |
| 2009 | 112.04 | 109.45 | 97.68 | 45.46 | 29.79 | 65.52 |
| 2010 | 317.23 | 144.89 | 45.67 | 91.10 | 33.48 | 36.75 |
| 2011 | 322.51 | 138.72 | 43.01 | 128.83 | 48.85 | 37.91 |
| 2012 | 833.84 | 428.91 | 51.43 | 151.15 | 57.42 | 37.99 |
| 2013 | 503.81 | 378.47 | 75.12 | 106.10 | 57.83 | 54.51 |
| 2014 | 588.64 | 261.96 | 44.50 | 115.20 | 48.98 | 42.52 |
| 2015 | 1341.67 | 771.85 | 57.52 | 228.81 | 89.39 | 39.07 |
| 2016 | 767.31 | 444.64 | 57.94 | 136.01 | 68.56 | 50.41 |
| 2017 | 1704.89 | 933.07 | 54.72 | 267.14 | 144.04 | 53.86 |
